# A quality assurance procedure to evaluate cone‐beam CT image center congruence with the radiation isocenter of a linear accelerator

**DOI:** 10.1120/jacmp.v11i4.3297

**Published:** 2010-07-02

**Authors:** Weiliang Du, James N. Yang, Eric L. Chang, Dershan Luo, Mary Frances McAleer, Almon Shiu, Mary K. Martel

**Affiliations:** ^1^ Departments of Radiation Physics The University of Texas M. D. Anderson Cancer Center Houston TX USA; ^2^ Departments of Radiation Oncology The University of Texas M. D. Anderson Cancer Center Houston TX USA

**Keywords:** CBCT, isocenter, IGRT, SBRT

## Abstract

A quality assurance (QA) procedure was developed to evaluate the congruence between the cone‐beam computed tomography (CBCT) image center and the radiation isocenter on a Varian Trilogy linac. In contrast to the published QA procedures, this method did not require a ball bearing (BB) phantom to be placed exactly at the radiation isocenter through precalibrated room lasers or light field crosshairs. The only requirement was that the BB phantom be in a stationary position near the radiation isocenter during the image acquisition process. The radiation isocenter was determined with respect to the center of the BB using a Winston‐Lutz test. The CBCT image center was found to have excellent short‐term positional reproducibility (i.e., less than 0.1 mm of wobble in each of the x (lateral), y (vertical), and z (longitudinal) directions) in 10 consecutive acquisitions. Measured over a seven‐month period, the CBCT image center deviated from the radiation isocenter by 0.40±0.12mm(x),0.43±0.04mm(y), and 0.34±0.14mm(z). The z displacement of the 3D CBCT image center was highly correlated (ρ=0.997) with that of the 2D kV portal image center. The correlation coefficients in the x and y directions were poor (ρ=0.66 and ‐0.35, respectively). Systematic discrepancies were found between the CBCT image center and the 2D MV, kV portal image centers. For the linear accelerator studied, we detected a 0.8 mm discrepancy between the CBCT image center and the MV EPID image center in the anterior‐posterior direction. This discrepancy was demonstrated in a clinical case study where the patient was positioned with CBCT followed by MV portal verification. The results from the new QA procedure are useful for guiding high‐precision patient positioning in stereotactic body radiation therapy.

PACS number: 87.55.Qr

## I. INTRODUCTION

Kilovoltage (kV) X‐ray images are increasingly used in radiation therapy to guide patient setup prior to treatment delivery. Compared to megavoltage (MV) portal images, kV images generally offer better visualization of bony anatomy, thus making it easier to align the patient to the planned position. Recent technical developments in kV cone‐beam computed tomography (CBCT) allow high‐resolution, three‐dimensional (3D) imaging of the patient within a reasonable acquisition time.^(^ ^1^ ^–^ ^3^ ^)^ Because its 3D capability provides enhanced visualization of bony structures and soft tissues, CBCT has gained significant clinical interest in a variety of radiation treatments.

The CBCT imaging system, like any linac accessory device used for patient positioning, must be subjected to rigorous spatial accuracy tests. Of these, the isocenter congruence test is particularly important. That is, the isocenter indicated on the CBCT images must coincide with the isocenter of the MV treatment beams (i.e., the radiation isocenter). Yoo et al.^(^ ^4^ ^)^ reported a widely used congruence test that employs CBCT images of a cube phantom that is placed at the radiation isocenter using room lasers and the light field crosshair.^(^ ^5^ ^–^ ^9^ ^)^ The accuracy of this approach is dependent upon the following conditions being met: (1) the room lasers and light field crosshair must be pre‐adjusted to the radiation isocenter; (2) the lasers and crosshair must remain stable over time; and (3) the phantom must be precisely aligned to the lasers and crosshair. In practice, these conditions are met with some uncertainties, typically in the range of 0.5 mm to 2 mm.^(^ ^10^ ^–^ ^12^ ^)^ The overall uncertainty from steps (1) to (3) combined can be comparable or exceed the amount of CBCT image center‐to‐radiation isocenter misalignment. Thus, extracting quantitative results on the CBCT image center accuracy may be difficult with this approach.

The purpose of this paper is to present a Winston‐Lutz (W‐L)^(^ ^13^ ^)^‐based method for a CBCT image center‐to‐radiation isocenter congruence test. The new method does not rely on the accuracy of the room lasers or light field crosshair. We use a stationary ball bearing (BB) phantom as the reference point to which both the radiation isocenter and the CBCT image center are located. The need for placing the phantom exactly at the radiation isocenter and the need for prior calibration of the room lasers or light field crosshair are eliminated. The phantom setup uncertainty is expected to be minimized with the present method, thus reducing the uncertainty in quantifying the CBCT image center congruence with the radiation isocenter.

## II. MATERIALS AND METHODS

**Figure 1:**
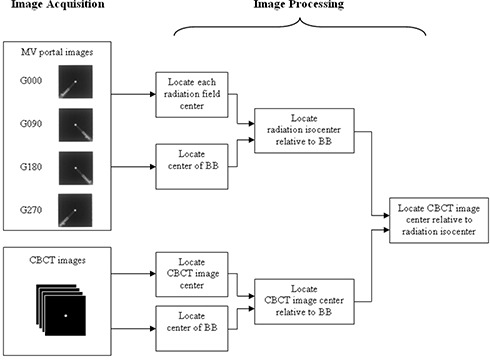
A work flow chart of the QA process.

A schematic view of our QA process is given in Fig. 1. The two major steps were image acquisition and image processing. In the first step, four MV portal images and one CBCT scan were acquired using a static BB phantom. In the second step, the MV portal images and the CBCT images were postprocessed to derive the displacement between the CBCT image center and the radiation isocenter.

### A. Phantom setup and image acquisition

**Figure 2:**
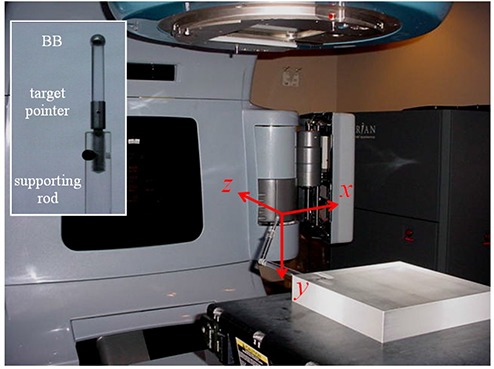
Experimental setup: a Varian Trilogy linac and a phantom containing a ball bearing (BB) on the tip (see inset) of a supporting rod. Also shown are the x‐y‐z directions of the DICOM coordinate system.

The phantom consisted of a Radionics target pointer (Integra Radionics, Burlington, MA), an acrylic supporting rod, and an acrylic base block (Fig. 2). The target pointer was secured through the supporting rod to the base block. The supporting rod was oriented obliquely so that it did not overlap with the x‐y‐z axes of the linac. At the tip of the target pointer was a tungsten BB (6.5 mm in diameter), wrapped with a thin plastic protective layer. The BB phantom was placed on the treatment table of a Varian Trilogy linac (Varian Medical Systems, Palo Alto, CA) and remained stationary during the entire data acquisition process. The same phantom was used for checking the coincidence between the 2D image graticules and the radiation isocenter.^(^ ^14^ ^)^ The center of the BB was used as a static reference point in the 3D space and was placed in the proximity of the linac isocenter using light field crosshair and room lasers. Given the routine monthly quality assurance (QA) procedures on the room lasers and light field crosshair, we were confident that the center of the BB was located within 2 mm of the radiation isocenter.

To locate the radiation isocenter, a simplified W‐L test was performed using the electronic portal imaging device (EPID). Four MV portal images of the BB phantom were acquired at the gantry rotation angles of 0°, 90°, 180° and 270°. These images are labeled as G000, G090, G180 and G270 in Fig. 1. The MV beams were a $10\times 10{\text{cm}}^{2}$ square field shaped with the Varian Millennium multileaf collimator (MLC). The couch and collimator rotation angles were kept at 0° for simplicity. A mechanical graticule was inserted in the gantry accessory tray if the graticule's image center accuracy was to be evaluated at the same time.^(^ ^14^ ^)^ Each MV portal image was a matrix of 1024×768 pixels, with a pixel size of 0.261mm×0.261mm at the isocenter distance (the physical pixel size on the detector plane was 0.391mm×0.391mm). The acquired MV images were saved and processed with an in‐house MATLAB (MathWorks, Natick, MA) program (see Section B below).

A CBCT scan of the phantom was acquired under a clinical image‐guided radiation therapy (IGRT) protocol, which was used in our spinal stereotactic radiation therapy (SRT) program for pretreatment patient setup. A half‐fan filter was used in the kV X‐ray beams. The kV detector panel was offset laterally by 14.8 cm during the CBCT scan to achieve a field‐of‐view of 45 cm in the x–y plane. Image reconstruction was performed with the clinical Varian on‐board imager (OBI) 1.4 software. The resulting 3D image dataset was a 512×512×160 matrix with a spatial resolution of 0.879mm×0.879mm×1.00mm in the lateral (x), vertical (y), and longitudinal (z) dimensions, respectively (see Fig. 2). These CBCT images were saved as 160 files in DICOM format for postprocessing (see section C below).

### B. Localization of radiation isocenter

**Figure 3:**
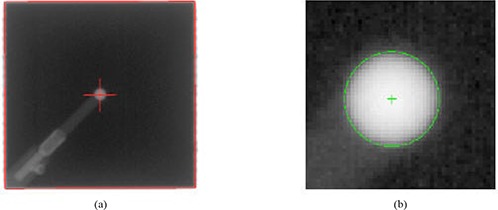
Localization of the radiation field center for one (G270 in of the MV portal images: (a) the radiation field edge and the center were detected automatically with a Hough transform‐based computer program; (b) the close‐up view shows the located BB and its center.

The radiation isocenter was determined by computer analysis of the MV portal images acquired at four cardinal gantry angles. First, the central axis of the radiation field in each MV portal image was located relative to the center of the BB (Fig. 3). A Hough transform (HT)‐based algorithm was used in this step to detect the radiation field edge as well as the edge of the BB.^(^ ^14^ ^,^ ^15^ ^)^ This algorithm was demonstrated to have subpixel accuracy in detecting circular or linear objects with varying sizes and shapes.^(^ ^14^ ^,^ ^15^ ^)^ Then the radiation isocenter was computed as a point in the 3D space that was closest to all four central axes. The resulting x, y, and z coordinates of the radiation isocenter were referenced to the center of the BB. Given that the precision of the image postprocessing was better than 0.1 mm,^(^ ^15^ ^)^ the uncertainty of the calculated radiation isocenter herein was dominated by the mechanical instabilities of the linac, which included the gravity‐induced gantry rotation inaccuracy (≤0.5mm radius sphere per Varian's specification) and the MLC leaf position inaccuracy.

### C. Localization of CBCT image center

To guide a radiation treatment, the CBCT images must contain a point defined within the image volume as the radiation isocenter of the linac. Such a point is referred to as the ‘CBCT image center’ or the nominal radiation isocenter in this study. The CBCT image center may deviate from the actual radiation isocenter because of errors in the calibration of the CBCT geometry or mechanical instability of the imaging system.

**Figure 4:**
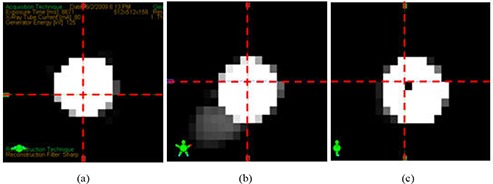
Axial (a), coronal (b), and sagittal (c) CBCT images of the BB phantom (high intensity area). Red crosshair represents the center of CBCT volume or the nominal radiation isocenter defined by the Varian CBCT software.

Immediately after the CBCT images were reconstructed, the cross‐sections of the BB were displayed in the Varian CBCT software along with the crosshair indicating the image center. Figure 4 shows a close‐up view of the CBCT image center relative to the BB. We found that the point defined by the crosshair was at the center of the CBCT volume. This was done by displaying the CBCT images in a MATLAB program, inserting a crosshair at the image volume center, and comparing it to the crosshair in the Varian CBCT software. In this study, the image center of a 512×512×160 CBCT volume was at the point between the 256th voxel and the 257th voxel in the x or y dimension, and between the 80th voxel and the 81st voxel in the z dimension.

**Figure 5:**
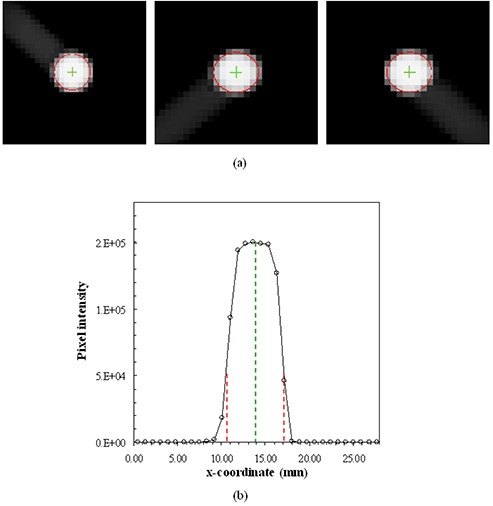
Localization of the BB center in 2D summation images. Images (a) were synthesized from the CBCT images by adding the pixel intensities along the z (left), y (middle), and x(right) directions, respectively. The edge (red circle or ellipse) and the center (green cross) of the BB were localized using the Hough transform algorithm. An image intensity profile (b) (circle points) along the x direction through the BB center was plotted with the located BB edge (red line) and the BB center (green line).

The center of the BB phantom was also located in the CBCT coordinates. First, a 2D summation image was formed by adding the CBCT image intensities along each of the x, y, z dimensions. The BB phantom appeared as a circle (or an ellipse, depending on the spatial resolution in each dimension) of high intensity at the central region of these summation images. Second, the centers of these circles or ellipses were located with subpixel accuracy through the HT algorithm.^(^ ^15^ ^)^ Figure 5 shows the detected edge and center of the BB in three summation images. Third, the x, y, and z coordinates of the BB center were averaged to obtain the final location of the BB center in the CBCT images. Finally, subtraction of the CBCT image center by the BB center, which were both in the CBCT image coordinates, gave the CBCT image center location relative to the BB center.

Once the radiation isocenter and the CBCT image center were localized in reference to the BB phantom, the 3D displacement between the CBCT image center and the radiation isocenter could be computed.

### D. Stability of CBCT image center

The short‐term and long‐term stabilities of the CBCT image center were studied. For short‐term stability, ten consecutive CBCT scans of the BB phantom were acquired while the phantom was static. The same CBCT protocol as described in Section A was used for all scans. The gantry was rotated clockwise in the odd‐numbered scans and counter‐clockwise in the even‐numbered scans. The CBCT image center location relative to the BB center was computed for each CBCT scan using the method described in Section C. For long‐term stability, the QA process described in Fig. 1 was repeated on a monthly basis. We monitored the CBCT image center‐to‐radiation isocenter congruence in a ten‐month period. For comparison, the 2D kV and MV portal imaging center‐to‐radiation isocenter congruence was also quantified in the same period.

### E. A clinical case study

The knowledge of the CBCT image center alignment is applicable to the clinical patient setup that involves CBCT image guidance. We report herein a case study for a patient undergoing spinal SRT. Prior to the SRT treatment, the patient had a C5‐C6 vertebrectomy and a spinal reconstruction with a titanium cage. A dose of 27 Gy was prescribed to treat the residual disease at C5‐C6 level in 3 fractions. The patient was immobilized in an Elekta BodyFIX (Elekta AB, Stockholm, Sweden) mold and a thermoplastic mask embedded in the mold. The CBCT‐guided patient setup was performed on the Varian Trilogy linac described above. Immediately following the CBCT setup, two orthogonal MV EPID images were taken to verify the patient's position. These images were analyzed by applying the measurement results from the monthly image center QA.

## III. RESULTS

**Figure 6:**
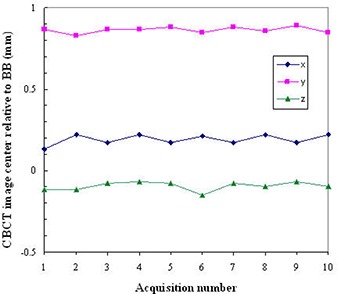
Short‐term stability of the CBCT image center. Ten CBCT scans were consecutively obtained using clockwise gantry sweeps (acquisition number 1, 3, 5, 7, 9) interleaved with counter‐clockwise gantry sweeps (acquisition number 2, 4, 6, 8, 10).

The short‐term stability of the CBCT image center is displayed in Fig. 6. The standard deviation of the CBCT image center wobble was 0.03 mm, 0.02 mm, and 0.03 mm in the x, y, and z directions, respectively. The range of the wobble was 0.09 mm (x), 0.06 mm (y), and 0.08 mm (z). There were small systematic displacements of the CBCT image center with the gantry rotation direction. This was most prominent in the x dimension. The displacement was 0.05 mm between the clockwise and counterclockwise CBCT scans.

**Figure 7:**
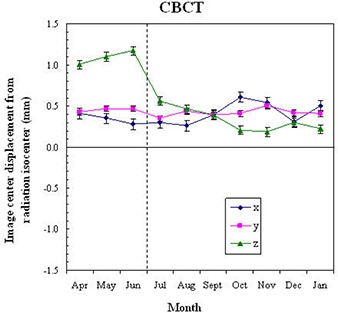
Long‐term stability of the CBCT image center alignment with the radiation isocenter. The vertical dashed line indicates that a QA adjustment of the kV detector panel position was done at the end of June. The error bars are ±2σ and σ is the standard deviation obtained from short‐term stability data (Fig. 6).

The long‐term stability of the CBCT image center is shown in Fig. 7. In a 10‐month period, the x, y, and z components of CBCT image center displacement from the radiation isocenter were 0.40±0.12mm,0.43±0.04mm, and 0.56±0.39mm, respectively. Because a greater than 1 mm displacement in z direction was observed from April to June, a mechanical adjustment of the kV detector panel was performed at the end of June. In the following seven‐month period (July to January), the z displacement was reduced to 0.33±0.14mm.

**Figure 8:**
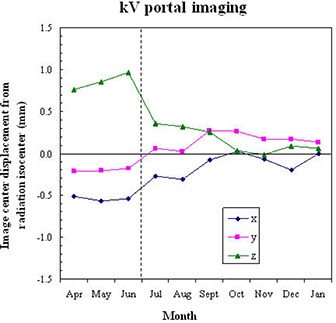
Displacement of the kV portal image center from the radiation isocenter, measured at a 0° and 90° kV source angles. The vertical dashed line indicates that a QA adjustment of the kV detector panel position was done at the end of June.

For comparison, the 2D OBI center displacement was evaluated using the kV portal images acquired at 0° and 90° source angles. Figure 8 shows the 2D OBI image center displacement from the radiation isocenter measured in the 10‐month period. The average displacements were 0.25±0.23mm,0.05±0.19mm, and 0.37±0.36mm in the x, y, and z dimensions, respectively. The z displacement (ΔZ) of the OBI from the radiation isocenter was highly correlated with the z displacement of the CBCT. The Pearson correlation coefficient was 0.997 between $\Delta Z_{\text{CBCT}}$ and $\Delta Z_{\text{OBI}}$. The correlation coefficients in the x and y directions were poor, only 0.66 between $\Delta X_{\text{CBCT}}$ and $\Delta X_{\text{OBI}}$ and ‐0.35 between $\Delta Y_{\text{CBCT}}$ and $\Delta Y_{\text{OBI}}$. $\Delta X_{\text{CBCT}},\;\Delta Y_{\text{CBCT}}$ were more stable than $\Delta X_{\text{OBI}},\;\Delta Y_{\text{OBI}}$, as evidenced by the smaller standard deviations in $\Delta X_{\text{CBCT}}$ and $\Delta Y_{\text{CBCT}}$. The maximum variations in $\Delta X_{\text{CBCT}}$ and $\Delta Y_{\text{CBCT}}$ in the 10‐month period were 0.35 mm and 0.15 mm, respectively, in contrast to 0.59 mm and 0.49 mm for $\Delta X_{\text{OBI}}$ and $\Delta Y_{\text{OBI}}$.

**Figure 9:**
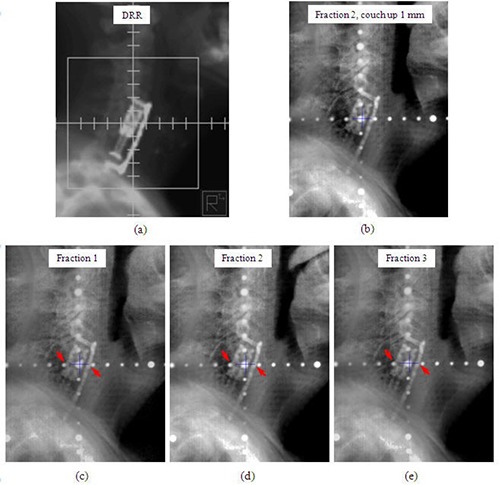
Image‐guided setup of a spinal SRT patient (3 fractions). The right‐lateral DRR reference image (a) was compared with the MV portal images (c), (d), and (e) acquired immediately following CBCT‐based patient alignment. There was an estimated 1 mm offset (red arrows) in the AP direction between DRR and MV portal images. A new MV portal image (b) was taken after applying a couch shift (raising couch 1 mm) in fraction 2. The blue crosses in the MV portal images were digital graticules superimposed by the record and verify software and were not relevant in this study.

Figure 9 illustrates a case study of image‐guided setup for a patient undergoing spinal SRT. Figure 9(c), (d), and (e) show the right lateral MV portals taken immediately after CBCT‐based couch adjustment. Compared to the digitally‐reconstructed radiograph (DRR) reference image(a), these MV portal images [(c), (d), (e)] were found to have approximately 1 mm offset in the anterior‐posterior (AP) direction between the titanium cage and the nominal radiation isocenter defined by the mechanical graticule. The offset was most eminent in fractions 2 and 3 by comparing the anterior and posterior edges of the titanium cage against the 1 cm tickmarks on the horizontal axis of the graticule. The offset was consistent in these MV portal images, which were acquired every other day. Based on the monthly QA analysis of the CBCT image center and the MV mechanical graticule, the CBCT image center was 0.4 mm posterior to the radiation isocenter, while the mechanical graticule center was 0.4 mm anterior to the radiation isocenter. Therefore, the CBCT image center was 0.8 mm posterior to the mechanical graticule center. This explained the approximate 1 mm offset in the AP direction in the MV verification portals. Figure 9(b) shows a new MV portal image taken in fraction 2 after applying a physician‐requested couch shift (i.e., the patient was raised up by 1 mm). In fractions 1 and 3, the couch was not adjusted based on the measured discrepancy between the CBCT image center and the MV mechanical graticule center. No apparent (≥0.5mm) discrepancy was found in the superior‐inferior or the lateral directions.

## IV. DISCUSSION

Numerous studies have reported QA procedures to check the congruence between the CBCT image center and the radiation isocenter.^(^ ^4^ ^–^ ^9^ ^)^ A common drawback in these studies is the reliance on room lasers and light field crosshairs in setting up a phantom to the radiation isocenter. The room lasers or light field crosshairs are calibrated according to the American Association of Physicists in Medicine (AAPM) TG‐40 requirements.^(^ ^12^ ^)^ However, the tolerances given in TG‐40 are 2 mm for the lasers and a 2 mm diameter for crosshair centering, which are insufficient for characterizing the small misalignments of CBCT or OBI image centers. Ali and Ahmad^(^ ^5^ ^)^ calibrated the lasers using the W‐L test, which is a standard QA method to locate the radiation isocenter in linac‐based radiosurgery. In our proposed method, the W‐L test was used for radiation isocenter localization. The need for calibrating the lasers or crosshairs was eliminated because the same BB phantom used in the W‐L test was imaged with the CBCT and 2D portal imaging systems. Furthermore, the BB did not have to be placed at the radiation isocenter, because the BB merely represented a reference point near the radiation isocenter. A strict requirement in our method was that the BB remain static during the entire imaging process. This technique is expected to reduce the phantom setup effort and to minimize the uncertainty in the resulting data.

Displacement of the CBCT image center from the radiation isocenter arises from mechanical inaccuracies of the linac and its on‐board imaging system. While inaccurate gantry rotation and collimator movement account for the wobble of the radiation field center, inaccurate kV source and kV detector movements cause the misalignment of the kV imaging system. There are systematic and random components of this misalignment.^(^ ^16^ ^)^ The systematic component should be minimized by implementing highly precise calibration procedures. Using our proposed method, we found that the measured displacements were reproducible, indicating that a significant portion of the misalignment was systematic and may be accounted for. For example, the x and y displacements of the CBCT image center were consistently about 0.4 mm (Fig. 7) over ten months. This 0.4 mm average displacement thus seemed to be systematic.

It is interesting to note that the CBCT image center misalignment correlates with the kV portal image center misalignment in the longitudinal (z) direction, not in the transverse (x and y) directions. In principle, if the OBI detector is longitudinally displaced from its ideal position, the image centers of both CBCT and 2D kV images are displaced accordingly. However, if the OBI detector is laterally displaced (in the detector plane and perpendicular to the z direction), the effects on the CBCT images and kV portal images are different. Because of the filtered back‐projection mechanism of CBCT image reconstruction, the detector lateral displacement is translated into blurring or ring artifacts in the CBCT images.^(^ ^17^ ^,^ ^18^ ^)^ The center position of an imaged object is not affected. In the kV portal images, however, the detector lateral displacement is simply reflected as a shift of the imaged object. In this sense, the CBCT image center is more robust against the translational errors in the OBI detector than the kV portal image center. This effect may explain the correlation observed between the CBCT and kV portal image centers.

When multiple imaging modalities are used for IGRT, different shift information may be derived for the same patient because each imaging modality represents the radiation isocenter differently.^(^ ^16^ ^)^ If such a discrepancy arises, the clinical dilemma is then deciding which imaging modality to trust. This is a particular concern in stereotactic treatments where submillimeter spatial accuracy is desirable. Figure 9 shows an example: although CBCT and EPID image centers are within 0.5 mm from the radiation isocenter, the apparent setup error in the EPID verification portal can be 1 mm. In this work, the discrepancy between CBCT and EPID portal imaging is resolved by the QA analysis of the congruence of the CBCT and EPID image centers with the radiation isocenter.

## V. CONCLUSIONS

We developed a fast and simple method to quantify the congruence of the CBCT image center with the radiation isocenter. The experimental error in phantom setup was eliminated in this method. The CBCT image center was compared against the radiation isocenter through a W‐L test. Computerized analysis revealed that the misalignment of the CBCT image center was highly reproducible in the short term as well as the long term, and could be quantified at submillimeter scale. The present method is useful for routine CBCT geometric calibration to ensure highly precise radiation dose is delivered to the patients.

## ACKNOWLEDGEMENTS

The authors are grateful to Drs. Peter Balter and Song Gao for help with initial image acquisition, and to Sue E. Moreau for editorial review of this manuscript.
